# Multi-Locus Typing of *Histomonas meleagridis* Isolates Demonstrates the Existence of Two Different Genotypes

**DOI:** 10.1371/journal.pone.0092438

**Published:** 2014-03-21

**Authors:** Ivana Bilic, Barbara Jaskulska, Rozenn Souillard, Dieter Liebhart, Michael Hess

**Affiliations:** 1 Clinic for Poultry and Fish Medicine, Department for Farm Animals and Veterinary Public Health, University of Veterinary Medicine Vienna, Vienna, Austria; 2 French Agency for Food, Environmental and Occupational Health Safety (Anses), European University of Brittany, Unit Epidemiology and Welfare in poultry and rabbit Ploufragan-Plouzane Laboratory BP 53 - 22440 Ploufragan, France; University of Hong Kong, China

## Abstract

*Histomonas meleagridis* is the aetiological agent of histomonosis or “blackhead disease”. Histomonosis is of special importance today, because there is no effective treatment to prevent its occurrence with considerable losses for the poultry industry. Despite its importance only a few molecular studies have yet been performed to investigate the degree of genetic diversity between different isolates of this parasite. In the present study a collection of well defined samples, previously shown positive for the DNA of *H. meleagridis*, was used to investigate genetic relatedness of the parasite. Samples originated from 25 turkey flocks collected in France between 2007 and 2010. Additionally, diagnostic samples, collected at our Clinic in Vienna, from different European countries and Azerbaijan, during 2010 to 2013 were included in the analyses. For the analysis three different genetic loci were analyzed: 18S rRNA, α-actinin1 and *rpb1* genes. To amplify partial sequences of α-actinin1 and *rpb1* genes, primers specifically targeting *H. meleagridis* were designed. Following PCR, the sequences of 18S rRNA, α-actinin1 and *rpb1* loci were analyzed. Phylogenetic analyses demonstrated separation of *H. meleagridis* isolates in two different clusters. The majority of isolates grouped within the cluster 1 and originated from different European countries. The cluster 2 was rare and predominantly found in samples originating from France. Considering that the genetic variability of clusters can be seen as two distinct genetic types we propose the term genotype instead of cluster.

## Introduction

*Histomonas meleagridis*, the aetiological agent of enterohepatitis termed histomonosis or “blackhead” disease [1], is a parabasalid protozoan parasite of the family Dientamoebidae/Protrichomonadinae (order Tritrichomonadida, class Tritrichomonadea) [2]. The family Dientamoebidae/Protrichomonadinae consists of additionally three other genera: *Dientamoeba*, *Protrichomonas* and *Parahistomonas*. The main characteristics of these protozoa are presence of single to two nuclei, the lack of infrakinetosomal body and comb-like structure in the mastigont, as well as the lack of undulating membrane and costa.

Histomonosis predominantly affects turkeys, but it is also more common in chickens and other gallinaceous birds [3], [4]. The disease is nowadays of special importance as the lack of therapeutics causes considerable economic losses within the poultry industry. The re-emergence of the disease gradually enforced research of this pathogen and recent years have seen some accumulation of molecular data on *H. meleagridis*. However, the degree of the genetic diversity between different isolates still seems poorly understood.

Several studies used the internal transcribed spacer-1 - 5.8S rRNA - internal transcribed spacer-2 (ITS1-5.8S-ITS2) region as a typing marker [5]–[8], which was shown to be very useful in determining genetic diversity of *Trichomonadidae* [9]–[13]. Yet, analyses with *H. meleagridis* indicated the existence of multiple sequence variants within a single isolate and a novel genotyping method termed “C-profiling” was introduced [5]. This method was based on the analysis of C-nucleotide patterns in sequencing chromatograms. A study of Hauck et al. [6] showed that C-profiles from different laboratories could not be compared. The authors supposed that this might be related to the presence of heterogeneous ITS-1 and ITS-2 sequences in a single clone, and demonstrated this by analyzing clonal *H. meleagridis* cultures. A recent investigation used ITS1-5.8S sequences for the analysis of different isolates and explained the occurrence of multiple sequence variants as a consequence of a mixed infection [8]. Investigations using protein-coding sequences for comparing different isolates struggled with the fact that the used sequences were not single copy genes and sequences originating from a single isolate were heterogeneous [14]–[16]. Therefore, these markers seemed not very useful for determining intra-species relationships. Nevertheless, position of *H. meleagridis*, as related to *Tritrichomonas foetus* could be seen in glyceraldehyde 3-phosphate dehydrogenase (GAPDH) and enolase trees which was concordant with phylogenetic studies on parabasalid microorganisms [2], [17]–[20].

In the present investigation genetic heterogeneity of *H. meleagridis* isolates was investigated by comparing phylogenetic data of partial 18S rRNA, alpha-actinin1 and *rpb1* gene sequences. Additionally, ITS1-5.8S rRNA-ITS2 sequences from different clonal cultures of *H. meleagridis* were analyzed to clarify the presence of sequence variants within the same isolate. Results of the analyses and applicability of each locus for sub-typing purposes are discussed.

## Results

### Analysis of 18S rRNA sequences

**Figure 1:**
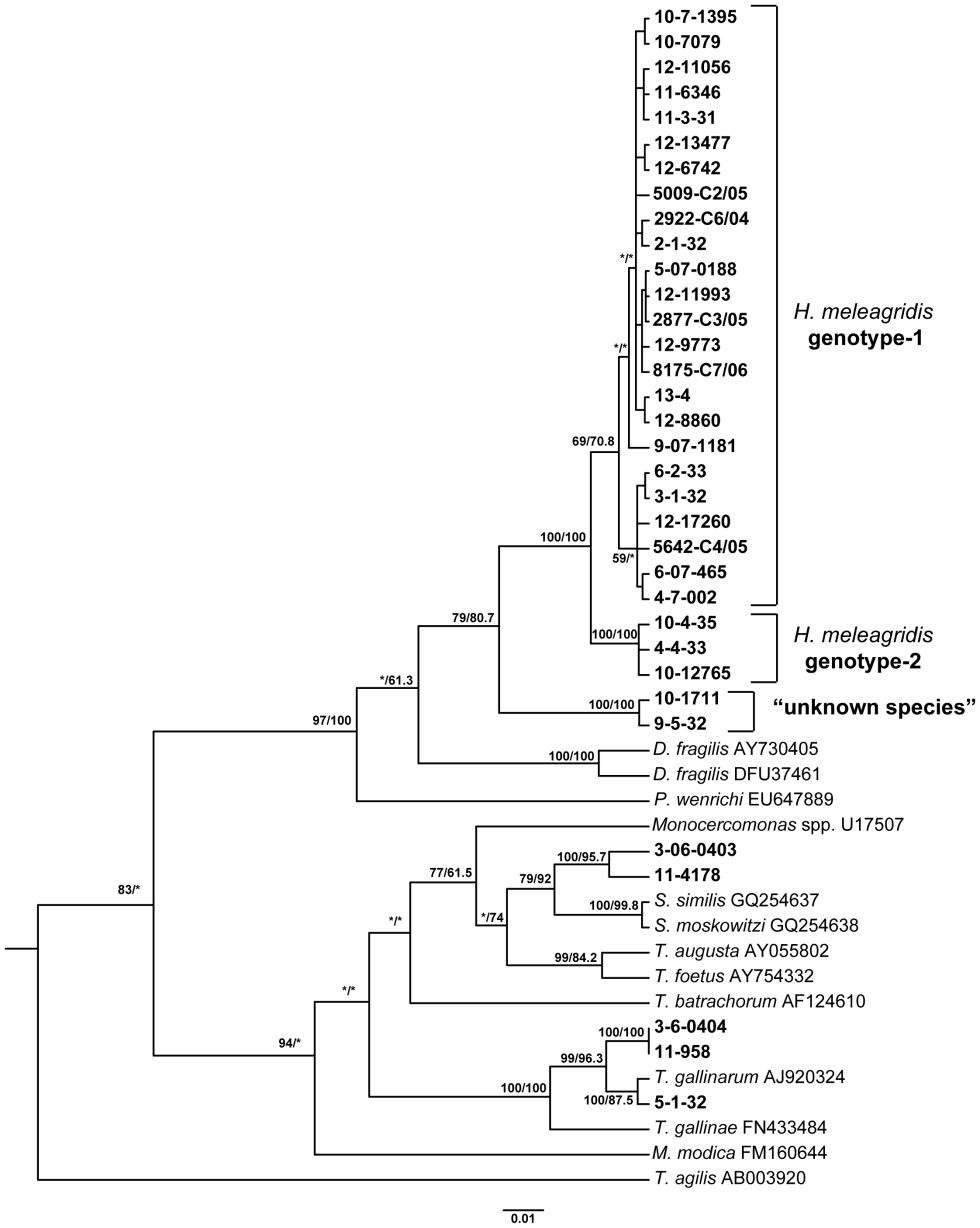
Phylogenetic tree of *H. meleagridis* isolates based on partial 18S rRNA sequences (approx. 544bp). For the analysis 47 sequences (sequence types) were used. The phylogenetic analysis was performed by applying separately maximum likelihood and neighbor-joining methods, with *Trichonympha agilis* as outgroup. The tree generated by maximum likelihood method is shown. Samples (N = 34) originating from this study are labelled bold. The robustness of the tree was determined by bootstrap re-sampling of the multiple–sequence alignments (1000 sets neighbor-joining/100 sets maximum likelihood). Values on the nodes are bootstrap support values indicated as percentages from neighbor-joining and maximum likelihood, respectively. Asterisks indicate nodes with bootstrap values below 50% or with a different topology. Branch lengths are proportional to sequence divergence and can be measured relative to the scale bar shown (bottom right). The scale represents nucleotide substitution per position.

Out of 256 samples PCR amplification and sequencing generated a total of 197 18S rRNA sequences, originating from 70 flocks (Table S1). Since the applied PCR amplified 18S rDNA from different protozoa, the BLAST search algorithm was used to identify the specificity of sequences to *H. meleagridis*. In case sequences proved to be fully identical with each other, only one of them was used for further analyses and was referred to as sequence type later in the text. In total 34 sequence types were identified. The analysis demonstrated 27 sequence types specific to *H. meleagridis* that shared 95.6–99.8% identity. In case a flock was sampled several times always a single sequence type was identified among all samples belonging to the same flock (Table S1). The relationship between *H. meleagridis* sequence types was determined by phylogenetic analysis using neighbor-joining and maximum likelihood methods. Both phylogenetic studies produced very similar tree topologies, with most branches being present in all trees with high statistical support. Analyses demonstrated distribution of sequence types in two major clusters, with 24 sequence types grouping within cluster 1 and 3 sequence types within cluster 2 (Figure 1). Consequently, cluster 1 was found in 109 samples from 58 flocks and cluster 2 in 56 samples from 7 flocks (Table S1).

The sequence identity within cluster 1 was 96.3–99.8% and within cluster 2 99.4–99.6%, whereas the identity between clusters ranged from 95.6% to 98%. All *H. meleagridis* sequences were shown to be closely related to *Parahistomonas wenrichi* and *Dientamoeba fragilis.* Since majority of isolates grouped to the cluster 1, it was broadly represented throughout Europe. In contrary, cluster 2 was predominantly found in France with the exception of a single isolate from Austria (Table S1).

Two sequences, detected in 3 samples from 2 different flocks, were shown specific to *H. meleagridis* according to the BLAST search. However, more detailed analysis demonstrated only 92.6–94.8% identity to *H. meleagridis* sequences. The sequence identities to the related protozoan species *P. wenrichi* and *D. fragilis* were 89.3% to 89.5% and 88.1 to 90.5%, respectively. The fact that these sequence types formed a separate cluster in the phylogenetic tree (Figure 1) and that their sequence identities were lower when compared to other *H. melagridis* isolates, indicated the detection of another species whose sequences are currently not available in the database.

Since the primers used to amplify 18S rDNA were not specific to *H. melagridis*, related protozoa like *Tetratrichomonas gallinarum* and *Simplicimonas* spp. could be identified in 26 samples, from 12 different flocks (Table S1). Out of these, *T. gallinarum* was detected in 16 samples, from 10 flocks, whereas *Simplicimonas* spp. was found in 12 samples, from 5 flocks. Detailed analysis showed that *Simplicimonas* spp. was represented with 2 and *T. gallinarum* with 3 sequence types (Table S1).

### Analysis of α-actinin1 sequences

PCR amplification using 256 samples resulted in only 88 α-actinin1 sequences, which were all specific for *H. meleagridis.* All generated sequences were analyzed. Generally, much lower genetic variability than with 18S rRNA sequences was detected (98.5–100% identity). Major differences were only present between sequences of isolates belonging to different clusters (98.5–98.6% identity). Analysis demonstrated that the cluster 1 sequences were found in 79 samples from 48 flocks whereas sequences from cluster 2 were amplified from 18 samples from 7 flocks (Table S1). Between representatives of two clusters twelve stable single nucleotide polymorphisms (SNPs) were detected (positions respective to FM200068: 219, 258, 264, 558, 579, 627, 789, 804, 813, 849, 850, 873, 924, 1080, 1092, and 1128). Only one SNP at position 850 lead to amino acid change (C to R). Similar to 18S rDNA analysis, only unique sequences were used for further analyses and referred to a sequence type later in the text.

**Figure 2:**
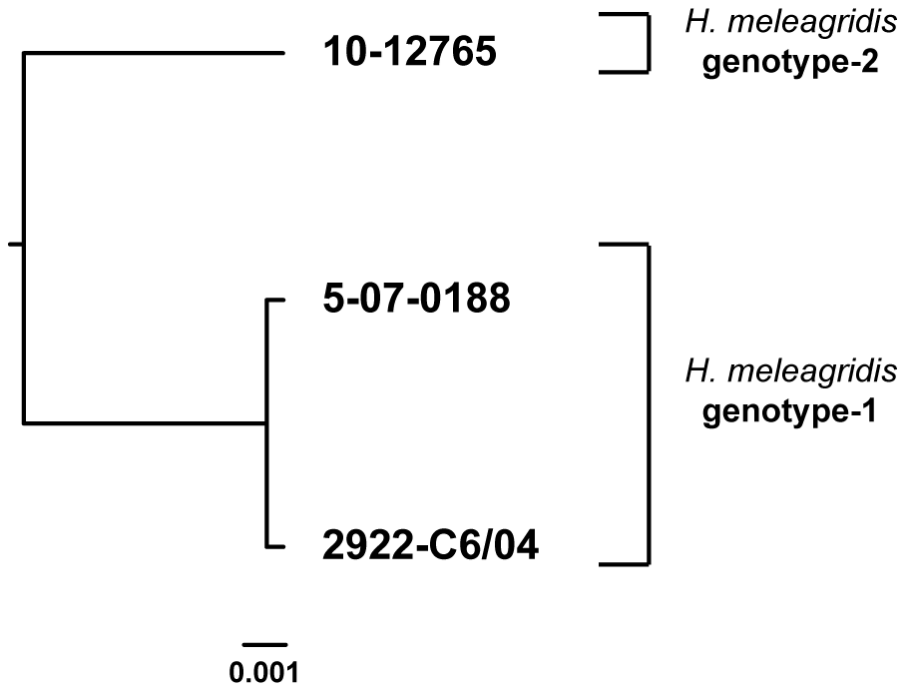
Phylogenetic tree based on partial α-actinin1 sequences of *H. meleagridis* (approx. 1048bp). The phylogenetic analysis was performed by applying separately maximum likelihood and neighbor-joining methods. The tree generated by maximum likelihood method is shown. Due to the low amount of analyzed sequences (N = 3) robustness of the tree by bootstrap re-sampling could not be determined. Branch lengths are proportional to sequence divergence and can be measured relative to the scale bar shown (bottom right). The scale represents nucleotide substitution per position.

Phylogenetic analysis confirmed the separation in two clusters (Figure 2), as already seen for 18S rRNA sequences. Sequences belonging to isolates of the same *H. meleagridis* cluster demonstrated very low genetic diversity (99.9% identity). Among cluster 1 sequences, only single SNP could be detected (T to C, position 850) and this one lead to amino acid change (C to R). Sequences obtained from isolates belonging to cluster 2 showed 100% identity. Hence, cluster 1 was represented with two sequence types and cluster-2 with only one.

Samples, whose 18S rRNA sequence types indicated a possibility of new species related to *H. melagridis*, gave no product with α-actinin1 PCR.

Eight samples from 4 flocks, in which 18S rRNA sequences were either specific for *T. gallinarum* or *Simplicimonas* spp., gave product in α-actinin1 PCR indicating a possible mixed infection (Table S1).

### Analysis of *rpb1* sequences

**Figure 3:**
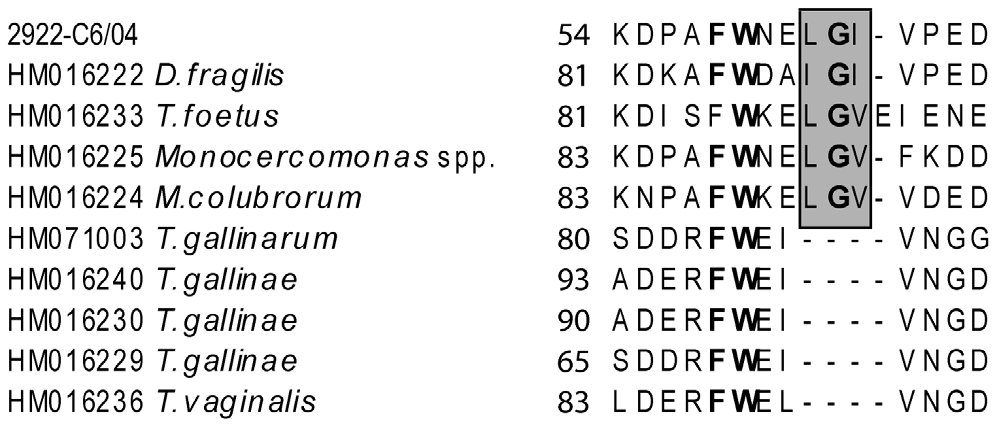
Amino acid alignment of *H. meleagridis* Rpb1 protein with Rpb1 proteins of Trichomonadea and Tritrichomonadea representatives. The alignment demonstrates a conserved insertion within region A of Rpb1 found only in Tritrichomonadea, present also in *H. meleagridis* Rpb1. The insertion is boxed, conserved amino acids are shown in bold, and gaps in the alignment are indicated by dashes.

The 2.93kb *rpb1* sequence of *H. meleagridis* was determined by using material from the clonal culture and degenerated primers. The *rpb1* identity of the sequence was proven by running the BLAST search algorithm. The generated sequence showed the highest identity to *rpb1* sequences of different protozoa. Protein alignment using deduced amino acid sequence demonstrated the conservation of the insertion in the region A of Rpb1 (Figure 3), found only in Tritrichomonadea [20]. Based on the 2.93 kb sequence, specific primers were designed to amplify 1.296 kb of the *rpb1* gene. Similarly to α-actinin1, the PCR amplified only sequences specific to *H. meleagridis* and out of 256 samples 46 sequences were generated (Table S1).

**Figure 4:**
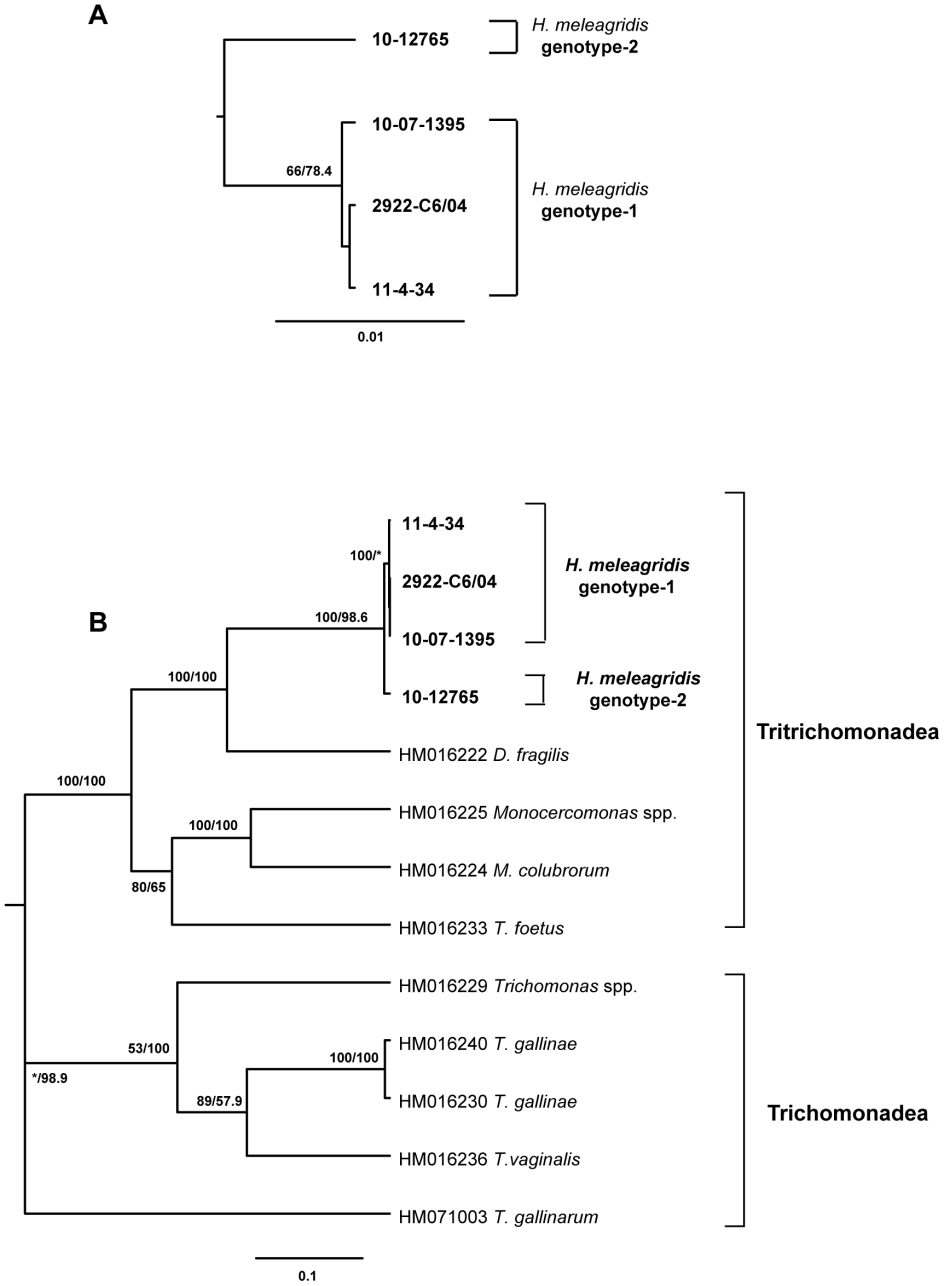
Phylogenetic trees based on partial *rpb1* sequences. (A) only *H. meleagridis* sequence types (N = 4) were used and the tree is based on 1242bp sequences. (B) *H. meleagridis rpb1* sequence types (N = 4) and sequences from different Tritrichomonadea (N = 4) and Trichomonadea (N = 5) available in the database were used; the tree is based on 759bp. Both analyses were performed with PHYLIP v3.68 software package applying separately neighbor-joining and maximum likelihood methods. Trees generated by maximum-likelihood method are shown. The robustness of the tree was determined by bootstrap re-sampling of the multiple–sequence alignments (1000 sets neighbor-joining/100 sets maximum likelihood). The values on the nodes are bootstrap support values indicated as percentages from maximum likelihood and neighbor-joining, respectively. Asterisks indicate nodes with bootstrap values below 50% or with a different topology. Branch lengths are proportional to sequence divergence and can be measured relative to the scale bar shown (bottom right). The scale represents nucleotide substitution per position.

Phylogenetic analysis using nucleotide sequences confirmed a clear separation of isolates into two clusters (Figure 4A). Cluster 1 sequences were detected in 35 samples from 32 flocks whereas sequences belonging to cluster 2 were identified in 10 samples from 6 flocks (Table S1). *Histomonas meleagridis* isolates demonstrated the closest relationship with *D. fragilis* and they clustered together with other Tritrichomonadea genera, like *T. foetus* and *Monocercomonas* strains (Figure 4B).

Genetic variability of sequences specific for *H. melagridis* was lower than the one observed in the analysis of the partial 18S rRNA sequence. However, *rpb1* sequences showed higher diversity than α-actinin1 sequences. Between representatives of clusters 1 and 2, seventeen stable SNPs could be noticed (positions respective to 2922-C6/04-complete: 613, 769, 826, 847, 940, 985, 1039, 1099, 1156, 1159, 1237, 1282, 1378, 1403, 1462, 1495, and 1510). Only one SNP, on position 1403, lead to amino acid change (S to P). Interestingly, this SNP was also present in sequence type 3 of cluster 1. Within cluster 1 three different sequence types were noticed. Most of the isolates had sequence type 1, represented in the phylogenetic tree with isolate 2922-C6/04. Sequence type 2 was rare, detected only in 2 isolates (11-2/4-31/35 and 12/11000) and differed from 2922-C6/04 in 1 position 952 (A to G). Sequence type 3 was detected in 9 isolates and is also characterized by one SNP: T to C at position 1403, the same SNP present in cluster 2 as well. Some samples with identical 18S rRNA sequences were shown to possess different *rpb1* sequence types, even though the latter locus displayed lower sequence variability among isolates belonging to the same cluster (Table S1).

Similar to the analysis of α-actinin1 sequences, samples whose 18S rRNA sequence types indicated a possibility of new species related to *H. meleagridis*, could not be amplified with *rpb1* primers specific for *H. meleagridis*. Three samples from 3 flocks, whose 18S rRNA sequences were shown specific for either *T. gallinarum* or *Simplicimonas* spp., resulted in a *H. meleagridis* specific *rpb1* PCR product, indicating a possible mixed infection (Table S1).

### Sequence analysis of the ITS1-5.8S-ITS2 locus

**Figure 5:**
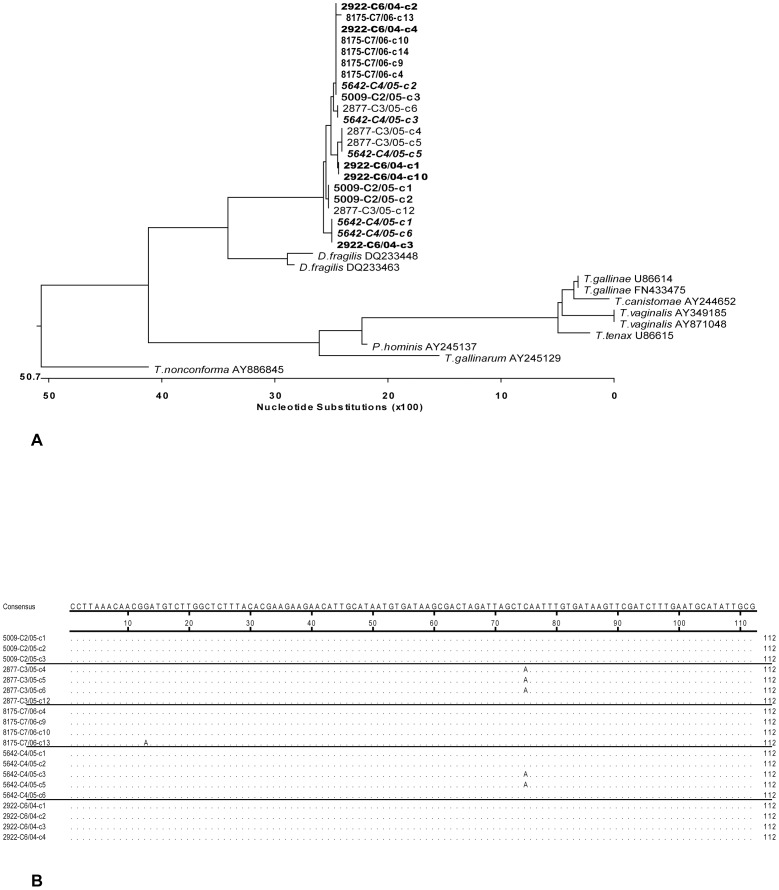
Analysis of ITS1-5.8S rRNA sequences from different H. *meleagridis* clonal cultures demonstrates the presence of multiple sequence variants within a single clone. (A) Phylogenetic tree based on the complete ITS1-5.8S rRNA-ITS2 region. Phylogenetic analysis of ITS1-5.8S rRNA-ITS2 region was performed using MegAlign application of Lasergene software (DNASTAR Inc.) by applying distance methods with default settings. Different clones are labelled with c and number, e.g. c1. (B) Sequence alignment of 5.8S rDNA region. Only sequence differences are shown, conserved nucleotides are designated as dots. Sequences originating from the same clonal culture are separated with lines.

The aim was to clarify the presence of multiple sequence variants in the ITS1-5.8S-ITS2 region within the same *H. meleagridis* isolate and evaluate this locus as a suitable marker for the analysis of intra-species variation. For that purpose, the advantage of *H. meleagridis* clonal cultures as well defined material was used. All ITS1-5.8S rRNA-ITS2 amplicons were cloned prior to sequencing and three to five different clones/culture were sequenced. The analysis demonstrated sequence heterogeneity in ITS1-5.8S rRNA-ITS2 region originating from a single clonal culture (Figure 5A). Furthermore, some of the sequences were shared among different clonal cultures, demonstrating that this locus cannot be used as unique sequence marker. Most variations were present in ITS1 and ITS2 regions, whereas 5.8S rDNA showed a higher level of sequence conservation within a single isolate. However, even in the most conserved 5.8S rDNA region single SNPs were detected among sequences originating from the same *H. meleagridis* clonal culture (Figure 5B).

## Discussion

The scope of the present study was to determine the level of genetic heterogeneity of *H. meleagridis* isolates by analyzing three different genetic loci, 18S rRNA, α-actinin and *rpb1* sequences. For that purpose, samples originating from different European countries and Azerbaijan were used. Analyses on all three loci demonstrated the separation of isolates into two major clusters. The observation that two genetically different clusters are resolved in all performed analyses supports the notion that in fact these clusters represent two different genotypes. In *D. fragilis*, the closest relative of *H. meleagridis*, two genotypes were described based only on 18S rRNA sequences. Sequence variation between these two genotypes differed depending on the length of the analyzed sequence, approximately 2% for 558bp and 4% for 1.7 kb [21], [22]. Here, it could be shown that the sequence variation between two clusters of *H. meleagridis* was in the similar range, 2–4.4% (for 600bp), which would also argue for using the term genotype instead of cluster. Therefore, we suggest that *H. meleagridis* isolates separate into two genotypes and we will use the term genotype instead of cluster further in the text. The majority of isolates grouped into *genotype 1*, whereas *genotype 2* appears to be rather rare. Due to the possibility of co-infections or other influences from the field the exact pathogenicity of isolates could not be determined. Indeed, in some samples the analysis revealed the existence of other protozoa such as *T. gallinarum* or *Simplicimonas* spp. together with *H. meleagridis*, which indicated a possible mixed infection. Furthermore, in two turkey flocks only *T. gallinarum* was detected, with one flock showing clinical signs of histomonosis and the other not (Table 1). The flock with clinical signs was analyzed as a single sample containing a pool of caeca from five birds, which possibly contributed to over dilution of *H. meleagridis* and resulted in the unsuccessful detection of this protozoan. Therefore, in order to pinpoint the agent that caused pathogenicity, a microorganism should be isolated and its virulence determined in infection experiments.

**Table 1:** Epidemiology data from French isolates collected in 2007–2010.

year	flock number	*H. meleagridis* genotype	age of birds (clinical signs)	number of visits		type of birds	initial number of birds	sex affected	initial mortality	
2007–2008	2	1	76 days	1		breeders	4841♀*	♀	27.5%	
2007–2008	4	1	42 days	1		meat	2900♂§ 2725♀	♂	50.3%	
2007–2008	5	1	41 days	1		meat	4500♂ 4500♀	♀ + ♂	32%♂ 16%♀	
2007–2008	6	1	74 days	1		breeders	313♂ 3390♀	♀	3%	
2007–2008	7	1	66 days	1		meat	5100♂ 4700♀	♀	10%	
2007–2008	8	1	33 days	1		meat	2750♀	♀	80%	
2007–2008	9	1	96 days	1		meat	4400♂ 4400♀	♂	2%	
2007–2008	10	1	126 days	1		breeders	3300♀	♀	0.7%	
2007–2008	62	1	85 days	1		meat	7956♂	♂	2%	
2007–2008	1	2	60 days	1		breeders	5408♀	♀	49.7%	
2007–2008	3	2	81 days	1		breeders	440♂ 4837♀	♀	4.1%	
2007–2008	32	negative *T.gallinarum* positive	n.d.	1		meat	4692♂ 4692♀	No clinical signs		
2009–2010	2	1	21	1	meat		9000♂ 7000♀	♂		9%
2009–2010	3	1	77	1	breeders		4880♀	♀		3.5%
2009–2010	5	1	100	1	breeders		2218♀	♀		25%
2009–2010	6	1	84	1	meat		4590♂ 4190♀	♂		18%
2009–2010	7	1	42	1	meat		4896♂ 4690♀	♂		1.5%
2009–2010	8	1	56	1	meat		6630♂	♂		20%
2009–2010	11	1	42	3	meat		6700♂	♂		21%
2009–2010	12	1	52	1	meat		6080♂ 5760♀	♂		17%
2009–2010	13	1	42	1	meat		3700♂ 3700♀	♂		1.6%
2009–2010	4	2	98	5	breeders		1147♂ 4240♀	♀		9.5%
2009–2010	9	2	50	6	meat		4681♂ 4161♀	♂		16%
2009–2010	10	2	84	8	breeders		405♂ 4416♀	♀		8%
2009–2010	14	negative$ *T.gallinarum* positive	40	1	meat		8000♂ 8000♀	♂		11%

*♀ =  females.

§ ♂ =  males.

$ this sample contained a pool of 5 caeca.

Each line represents the data from a single flock.

The degree of heterogeneity of *H. meleagridis* isolates belonging to a single genotype differed depending on the analyzed genetic locus. The analysis of 18S rRNA sequences demonstrated a clear genetic diversity of *H. meleagridis* isolates within each genotype. Interestingly, even though data on genetic diversity of *D. fragilis* isolates seem very similar, clear heterogeneity of *D. fragilis* isolates belonging to a single genotype was not reported and *D. fragilis* was even considered a clonal species [21], [22].

Analyses based on two protein coding genes, α-actinin1 and *rpb1*, confirmed the separation of *H. meleagridis* isolates into two genotypes. However, these analyses showed discrepancy concerning the genetic heterogeneity within a single genotype. The analysis of *rpb1* locus demonstrated moderate heterogeneity within *genotype 1*, as fewer sequence types than in 18S rDNA analysis were determined. Analysis of α-actinin1 sequences demonstrated extremely low sequence variation within a single genotype, with 2 sequence types for *genotype 1* and a single one from *genotype 2*. Discrepancy in the outcome of these analyses could be explained by the fact that the sequence conservation of each genetic locus is variable, and it is often stronger for protein coding sequences. Furthermore, repetitive nature of 18S rDNA region within a genome could favour micro-heterogeneity among different copies present within the genome. Gerbod et al. [17] reported limited heterogeneity of rDNA region from *H. meleagridis*, as they sequenced the ends of 18S rRNA coding region (350bp from 5′-end and 500bp from 3′-end) from several clones of a PCR product. However, since the rest of the sequence was determined only once it is not clear whether such trend is present throughout the sequence. Later, Mantini et al. [18] also detected micro-heterogeneity in the 18S rDNA of *H. meleagridis* when sequencing two clones of PCR products that amplified the 1.56 kb of 18S rDNA region. However, authors of both studies commented that amplification errors could not be excluded when explaining the nature of micro-heterogeneity in 18S rDNA. It is important to note that the *H. meleagridis* material used for both studies hasn’t come from a culture generated from a single protozoal cell, a so called clonal culture; therefore a possibility of a mixed infection could not be excluded. Since 18S rDNA is generally present in multiple copies within a genome, this still leaves the possibility that certain level of micro-heterogeneity is present throughout this locus, which could be more accurately elaborated by deep sequencing technique. In the present analysis we used a central region of 18S rRNA sequence and all samples were sequenced directly without cloning. No sequence ambiguities (double peaks in the chromatograms) were identified in any of the directly sequenced PCR products. Moreover, a single sequence type was detected among all samples belonging to the same flock. All of this indicates a uniformity of generated 18S rRNA sequences from an isolate and argues for exploitation of this locus as a sub-typing tool.

The other two genetic loci used in the present investigation, α-actinin and *rpb1*, demonstrated lower genetic diversity compared to 18S rDNA, which can be explained by their protein coding nature. Both, α-actinin and *rpb1* seem to be single copy genes because in all samples they were directly sequenced without a cloning step and no sequence uncertainties were detected when analysing chromatograms. Such property is important when a genetic locus is considered to be used for sub-typing, supposing that the level of sequence conservation is not too high. The *rpb1* locus displays moderate sequence diversity within a single cluster and provides additional information on isolates. This became more evident when some isolates that were classified as identical with 18S rDNA displayed different *rpb1* sequences. Therefore, data presented here advocate the use of the *rpb1* locus as a sub-typing tool for *H. meleagridis*, despite its’ lower genetic diversity. The Rpb1 was already shown as useful marker for phlyogenetic analysis of parabasalids at various levels of taxonomic resolution, i.e. isolate, species, genus and upward [20]. The discrepancy in fine classification of isolates depending whether the 18S rDNA or the *rpb1* loci were used, could be explained by the fact that in the *rpb1* analysis much longer sequences were used. The amplification of longer 18S rDNA region was not as efficient when field samples were used, therefore a PCR assay amplifying shorter segment was employed. Alpha-actinin1 locus showed the lowest sequence diversity with clear sequence variations present only when representatives of two genotypes were analyzed. These features clearly argue against the use of this locus as a marker for sub-typing *H. meleagridis* isolates.

Analysis of 18S rRNA locus identified two sequences of an unknown species related to *H. meleagridis*, *D. fragilis* and *P. wenrichi*, which formed a separate cluster in phylogenetic analysis. *Histomonas*-specific PCRs amplifying α-actinin1 and *rpb1* sequences were negative; therefore no further sequences were obtained. The identity of this species is unknown, as matching sequence data are not available in the database. All analyzed samples, except the clonal cultures, were not cultured and so it was not possible to inspect the morphology of this protozoan. Consequently, it remains to speculate to what species these sequences belong. The last new classification of parabasalia based on the existing ultra-structural and molecular phylogeny data, designates four genera within the family Dientamoebidae: *Dientamoeba*, *Histomonas*, *Parahistomonas* and *Protrichomonas* [2]. Sequence data for three of these genera are available and only sequences of *Protrichomonas* are still not identified. Whether these 18S rRNA sequences really belong to *Protrichomonas* spp., or to another unknown relative still remains to be investigated.

The ITS1-5.8S rRNA-ITS2 region is commonly used to investigate polymorphisms between organisms, as ITS regions are non-coding and evolve rapidly. Different previous investigations found this region useful for molecular phylogeny of trichomonadid protozoa [12], [13]. The present study demonstrated the existence of multiple sequence variants of ITS1-5.8S rRNA-ITS2 region within a single cell, by analyzing different clonal cultures. Further confusion appeared with the fact that same sequences are shared among different clonal cultures. All this made the assignment of a certain sequence as the unique marker of an isolate impossible and classified this marker as unsuitable for sub-typing purposes. However, it should be noted that all clonal cultures used in the analysis belong to genotype 1; therefore it was not possible to determine whether this locus would be sufficient for the differentiation of the two genotypes. Interestingly, similar results of variable ITS1-5.8S rRNA-ITS2 regions within a single isolate were already reported for *D. fragilis* [23], suggesting that this feature might be a characteristics of Dientamoebidae. It would be interesting to investigate whether other genera of this family display similar features.

Earlier *H. meleagridis* sub-typing studies already indicated sequence variations of ITS1-5.8S rRNA-ITS2 region within a single *H. meleagridis* isolate [5], [6], and that lead to introduction of a novel method named C-profiling. C-profiling is based on the analysis of only C-nucleotide peaks within the chromatogram which simplifies the analysis. The method seems straightforward, easy to apply, and it was used for sub-typing of *H. meleagridis* and *D. fragilis* [5], [6], [24]. However, the validity of the method as a general sub-typing tool seems rather questionable. Several points argue for such an opinion. Data comparison between two different laboratories seems at the moment not possible and the evaluation of different C- profiles looks rather intuitive. Furthermore, it was never reported whether the method is reproducible when different sequencing companies or machines are used to generate chromatograms. It was only reported that identical C-profiles were obtained by several independent analyses of a sample within the same laboratory [24]. The obvious drawback stems from the fact that the method is not standardized and the “*in-silico*” support, as database and software for the analysis, is not developed. Therefore at the moment it seems that C-profiling, if at all, could only be used as a sub-typing method within a laboratory.

Conclusively, by analyzing different genetic loci the present study demonstrated for the first time the separation *H. meleagridis* isolates into two genotypes. *Genotype 1* seemed to be broadly represented in Europe, whereas *genotype 2* was rare. Genetic variation within a single genotype differed depending on the locus that was used. Both 18S rRNA and *rpb1* loci proved to be useful for determining genetic variation of *H. meleagridis* isolates, and should optimally be combined for detailed analyses. In contrary, α-actinin1 locus was shown not to be suitable due to high sequence conservation among representatives of a single genotype. The sequence analysis of ITS1-5.8S rRNA-ITS2 locus using different *H. meleagridis* clonal cultures confirmed the heterogeneity of sequences within a single cell. We advise against the usage of ITS1-5.8S rRNA-ITS2 locus for purposes of molecular sub-typing of *H. meleagridis*, since the presence of sequence variants within a single isolate of this protozoan designate this marker as unsuitable.

Future studies should concentrate on investigating the biological relevance of the two genotypes and whether the noticed *genotype 2* in France contributes to the high incidence of histomonosis in that country.

## Materials and Methods

### Source of the samples

Diagnostic samples (N = 39) previously positive for *H. meleagridis* collected at our Clinic between 2010 and 2013 were included in the analyses. The samples originated from Austria, Azerbaijan, Denmark, France, Germany, Hungary, Netherlands, Poland and the United Kingdom. Furthermore, a set of 211 caecum samples from 25 different turkey flocks collected in France between 2007 and 2010 were analysed (Table 1). For this, 5 birds per flock were sampled during one visit. Except for a single flock each sampled bird was treated as a separate sample (Table S1). Some (N = 4) flocks were visited on multiple occasions, always in weekly intervals.

Six clonal cultures established at our Clinic were also included in the analysis. Establishment of clonal cultures and *in vitro* propagation were performed as previously published [25]. The material used for establishing clonal cultures originated from field cases in Austria. A detailed list of all samples used in the present study is displayed in Table S1.

Diagnostic samples collected at the Clinic for Poultry and Fish Medicine were part of the veterinary medicine diagnostic investigation and therefore do not need an ethical approval. The samples originating from the French study were also part of the veterinary medicine diagnostic investigation of histomonosis and therefore do not need an ethical approval.

Samples from the French study were collected from turkey farms by veterinarian of French Agency for Food, Environmental and Occupational Health Safety (Anses), and the participation was based on the owner’s agreeing to take part in the study.

None of the samples was received from the third party.

### PCR and sequencing

DNA was extracted by employing the DNeasy Blood & Tissue Kit (Qiagen, Vienna, Austria) following manufacturer’s instructions. All PCRs were performed in 25μl reaction by using HotStar Taq Master Mix Kit (Qiagen, Vienna, Austria) and 0.4μM of each primer. Negative controls (without template) were used throughout the specimen preparation and PCR progress. In addition, PCR negative controls were employed to demonstrate that PCR mastermix was free of any contamination. For amplification of 603bp sequence of 18SrRNA gene, primers 18S-F and 18S-R (Table 2) were used. The amplification of 1.160kb of α-actinin1 sequence was performed with actinin1-CH-EFhF and actinin1-CH-EFhR primers (Table 2). Thermo-cycling conditions for both PCRs were: one cycle of 95°C for 15 minutes; 40 cycles of 95°C for 30 seconds, 53°C for 30 seconds and 72°C for 1 minute; followed by final elongation step at 72°C for 10 minutes. For amplification of 1.296kb of *rpb1* sequence primers HmRpb1-short2F and HmRpb1-short2R (Table 2) were used. Primers were based on the partial sequence of *H. meleagridis rpb1* gene. Detailed description of how *rpb1* sequence was determined is given in File S1. Reaction conditions were: one cycle of 95°C for 15 minutes; 40 cycles of 95°C for 30 seconds, 51.7°C for 30 seconds, 72°C for 90 seconds; followed by final elongation step at 72°C for 10 minutes.

**Table 2:** Primers used in this study.

primer name	Sequence 5′ to 3′	origin
HmRpb1-F2	GTN ATH TTY AAY CGD CAR CC	this study*
HmRpb1-R2	CTT CVA TGT TYT GYT GAC C	this study*
HmRpb1-F3	TTG CTA CTG TAG GTC AAC A	this study
HmRpb1-R3	ATA ACT GCA TGA TGT CCC ATC A	this study
Rpb1-F1	GAG TGT CCA GGN CAY TTY GG	[20]
Rpb1_mod_R2	AAT TGT GTA GMT GGT TCA CC	this study*
HmRpb1-longF	ATT GCG TAT GCC ACA AAT G	this study
HmRpb1-longR	ATA GCA CCT ACC ATT TCA C	this study
Hm-Rpb1-shortF	GGA TCA CTT ACA CGA CAA GAC	this study
HmRpb1-shortR	TCG TTG TTC GAC TTC TTG C	this study
HmRpb1-short2F	AGA TCC AGA ACG ATC TCA TCC-	this study
HmRpb1-short2R	TGA CTT AAG AAA TCT CGT GCC	this study
18S-F	GCA GTT AAA ACG CTC GTA GTC-3’	this study
18S-R	AAC GCT AGA CAG GTC AAC CC	this study
actinin1-CH-EFhF	GCA AAA CAC CTT GCC ACT AAG	this study
actinin1-CH-EFhR	GCA CGC TTC TCT TCA AGT TC	this study
TFR2	CGG TAG GTG AAC CTG CCG TTG G	[13]
TFR1	TGC TTC AGT TCA GCG GGT CTT CC	[13]

* design based °n the Rpb1 alignment of different parabasalid sequences.

Amplification of approximately 350bp ITS1-5.8S rRNA-ITS2 region was performed by using TFR1 and TFR2 primers (Table 2) with the cycling conditions: 95°C for 15 minutes, 40 cycles of 95°C for 30 seconds, 55°C for 1 minute, 72°C 1 minute and final elongation step 72°C for 10 minutes. Amplification products (25 μl) were electrophoresed in a 1.5% Tris acetate-EDTA-agarose gel, stained with ethidium bromide and visualized under UV light (Biorad Universal Hood II, Bio-Rad Laboratories, California, USA). Fragment sizes were determined with reference to a 100bp ladder (Invitrogen, Life Technologies, Austria). Standard precautions were applied to avoid PCR contamination. PCR reagents were aliquoted; aerosol barrier tips, dedicated pipette sets, laminar flow hoods and separate laboratory areas were used for each step of the procedure. PCR products of the expected sizes were excised from the gel and purified using the QIAquick Gel Extraction Kit (Qiagen, Vienna, Austria) according to the manufacturer’s instructions. Direct fluorescence-based sequencing was performed by LGC Genomics GmbH (Berlin, Germany) using the PCR primers. The exception were ITS1-5.8S rRNA-ITS2 amplicons, which were cloned to TOPO vector (TOPO TA Cloning Kit for Sequencing, Invitrogen, Life Technologies, Austria) prior to sequencing. Fluorescence-based sequencing of three to five clones was performed by LGC Genomics GmbH (Berlin, Germany) using M13 primers. All sequences were deposited to the EMBL database [EMBL: HG008073-HG008105, HG008107-HG008112].

### Sequence analyses

Assembly and analyses of sequences, the nucleotide and amino acid alignments were performed with Acccelrys Gene, version 2.5 (Accelrys, San Diego, CA), Lasergene (DNASTAR Inc.) and ClustalX v2.1 [26] software. Primer binding sites were excluded from sequences used in the analyses.

Phylogenetic analysis of 18S rDNA (aprox. 544bp), α-actinin1 (819bp) and *rpb1* (759bp and 1242bp) nucleotide sequences were performed with the aid of PHYLIP package version 3.68 [27] using distance and maximum likelihood methods. For all PHYLIP applications default settings were used if not otherwise specified. Bootstrap re-sampling analyses of 1000 replicates for distance trees and 100 replicates for maximum likelihood trees were performed with the SEQBOOT program to prove the stability of the trees. For analyses with nucleotide sequences, distance matrices were calculated by using DNADIST application with the Kimura 2-parameter model and translation/transversion ratio of 2.0. Maximum likelihood analysis was performed by using DNAMLK application. The final phenogram was produced by DNAMLK where the tree obtained by CONSENSE was implemented as user tree. The graphical output was generated using DRAWGRAM program.

Phylogenetic analysis of ITS1-5.8S rRNA-ITS2 region was performed using MegAlign application of Lasergene software (DNASTAR Inc.) with default settings.

## Supporting Information

File S1 Cloning of rpb1. Detailed description of *rpb1* cloning strategy.
(DOCX)

Table S1 List of all isolates used in the study.
(DOCX)
