# Supplementary material for: Multi-Locus Typing of Histomonas meleagridis Isolates Demonstrates the Existence of Two Different Genotypes
Source: PLoS One. 2014 Mar 21;9(3):e92438. doi: 10.1371/journal.pone.0092438 (PMC3962415; doi:10.1371/journal.pone.0092438)
Supplement: File S1 — Cloning of rpb1. Detailed description of rpb1 cloning strategy. (DOCX) [file pone.0092438.s001.docx]

**Supporting information- File S2**

**File S2 Cloning of rpb1**

DNA obtained from the clonal culture *H. meleagridis* /Turkey/Austria/2922-C6/04 was used as template for all PCR reactions. The partial sequence of *H. meleagridis* *rpb1* gene was determined in several steps. DNA was extracted by employing the DNeasy Blood & Tissue Kit (Qiagen, Vienna, Austria) following manufacturer’s instructions. All PCRs were performed in 25µl reaction by using HotStar Master Mix (Qiagen, Vienna, Austria) and 0.4µM of each primer. Negative controls (without template) were used throughout the specimen preparation and PCR progress.

Initially, degenerated primers HmRpb1-F2 and HmRpb1-R2 (Table 2) were used to amplify the central 1.015kb of the gene, by applying following cycling conditions: 95°C for 15 minutes; 40 cycles of 95°C for 1 minute, 45°C for 1 minute, 50°C for 1 minute, 55°C for 1 minute, 72°C for 2 minutes + 6 seconds/cycle; final extension at 72°C for 10 minutes. The PCR fragment was gel purified by using QIAquick Gel Extraction Kit (Qiagen, Vienna, Austria) according to the manufacturer’s instructions and cloned into the TOPO vector (TOPO TA Cloning Kit for Sequencing, Invitrogen, Life Technologies, Austria). Fluorescence-based sequencing of two clones was performed by LGC Genomics using M13 primers. The identity of the sequence was controlled by running the BLAST algorithm. Based on this sequence, specific primers HmRpb1-F3 and HmRpb1-R3 (Table 2) were designed to amplify the N- and C- terminal part. For the N-terminal part, 1.154kb was amplified with primers Rpb1-F1 and HmRpb1-R3 (Table 2). The 1.716kb of the C-terminal part was amplified by using HmRpb1-F3 and Rpb1_mod_R2 (Table 2). Cycling conditions for both PCRs were: 95°C for 15 minutes; 40 cycles of 95°C for 1 minute, 45°C for 1 minute, 50°C for 1 minute, 55°C for 1 minute, 72°C for 2 minutes + 6 seconds/cycle; final extension at 72°C for 10 minutes. In order to verify the sequence, the *rpb1* gene was re-amplified by using HmRpb1-longF and HmRpb1-longR (Table 2) with the following cycling conditions: 95°C for 15 minutes; 40 cycles of 95°C for 30 seconds, 52°C for 30 seconds, 72°C for 3 minutes + 6 seconds/cycle; final extension 72°C for 10 minutes. The obtained 2.968kb fragment was directly sequenced by using HmRpb1-longF, HmRpb1-longR, Hm-Rpb1-shortF and HmRpb1-shortR (Table 2). All PCR products were fractionized by agarose gel electrophoresis (1% agarose in Tris-acetate EDTA), stained with ethidium bromide and visualized under UV light (Biorad Universal Hood II, Bio-Rad Laboratories, California, USA). Fragment sizes were determined with reference to a 1kb ladder (Invitrogen, Life Technologies, Austria). PCR products of the expected sizes were excised from the gel and purified using the QIAquick Gel Extraction Kit^®^ (Qiagen, Vienna, Austria) according to the manufacturer’s instructions. Direct fluorescence-based sequencing was performed by LGC Genomics using the PCR primers if not stated otherwise.

The obtained 2.930kb *rpb1* sequence (without primers) was submitted to the EMBL database [EMBL: HG008112].
