# Supplementary material for: Multi-Locus Typing of Histomonas meleagridis Isolates Demonstrates the Existence of Two Different Genotypes
Source: PLoS One. 2014 Mar 21;9(3):e92438. doi: 10.1371/journal.pone.0092438 (PMC3962415; doi:10.1371/journal.pone.0092438)
Supplement: Table S1 — List of all isolates used in the study. (DOCX) [file pone.0092438.s002.docx]

**Supporting information**

**Table S1 List of all isolates used in the study**

| **flock name** | **country of origin** | **type of birds** | **year** | ***H. meleagridis* genotype** | **number of samples** | **18S accession number** | **α-actinin1 accession number** | ***rpb1* accession number** |
| --- | --- | --- | --- | --- | --- | --- | --- | --- |
| 10/1711 | Hungary | breeders | 2010 | unknown species | 1 | **HG008099** | n.d.* | n.d. |
| 10/7079 | Austria | laying chicken | 2010 | I | 1 | **HG008073** | HG008108 | n.d. |
| 10/9654 | Germany | meat turkey | 2010 | I | 1 | HG008086 | HG008108 | HG008112 |
| 10/9655 | Germany | meat turkey | 2010 | I | 1 | HG008090 | HG008108 | n.d. |
| 10//11616 | Germany | unkown^$^ | 2010 | I | 1 | HG008090 | n.d. | n.d. |
| 10/12765 | France | turkey^#^ | 2010 | II | 1 | **HG008095** | **HG008107** | **HG008109** |
| 10/13399 | Austria | organic young chicken | 2010 | I | 1 | n.d. | HG008108 | n.d. |
| 10/15699 | Germany | turkey^#^ | 2010 | I | 1 | HG008086 | HG008108 | n.d. |
| 11/958 | Austria | unknown | 2011 | *T. gallinarum* | 1 | **HG008101** | n.d. | n.d. |
| 11/4178 | Austria | laying chicken | 2011 | *Simplicimonas spp.* | 1 | **HG008105** | n.d. | n.d. |
| 11/6346 | Austria | organic laying chicken | 2011 | I | 1 | **HG008087** | HG008108 | n.d. |
| 11/6303 | Netherlands | meat turkey | 2011 | I | 1 | HG008074 | HG008108 | n.d. |
| 11/7276 | Germany | laying chicken | 2011 | I | 1 | HG008076 | n.d. | n.d. |
| 11/7281 | Germany | laying chicken | 2011 | I | 1 | HG008074 | HG008108 | n.d. |
| 11/8622 | Germany | laying chicken | 2011 | I | 1 | HG008074 | n.d. | n.d. |
| 11/9225 | Austria | bantam chicken | 2011 | I | 1 | HG008074 | HG008108 | n.d. |
| 11/10299 | Germany | broiler breeder | 2011 | I | 1 | HG008074 | HG008108 | HG008112 |
| 11/11085 | Germany | laying chicken | 2011 | I | 1 | HG008086 | n.d. | n.d. |
| 11/12040 | Germany | laying chicken | 2011 | I | 1 | HG008090 | n.d. | n.d. |
| 11/12699 | Austria | laying chicken | 2011 | I | 1 | HG008098 | n.d. | n.d. |
| 11/12778 | Poland | chicken^#^ | 2011 | I | 1 | HG008086 | n.d. | n.d. |
| 11/12779 | Poland | chicken^#^ | 2011 | I | 1 | HG008090 | HG008108 | n.d. |
| 12/6742 | Austria | meat turkey | 2012 | I | 1 | **HG008088** | HG008108 | HG008112 |
| 12/8860 | Germany | meat turkey | 2012 | I | 1 | **HG008090= EU647884** | HG008108 | HG008112 |
| 12/9417 | Azerbaijan | turkey breeder | 2012 | I | 1 | HG008086 | n.d. | HG008110 |
| 12/9558 | Hungary | breeder | 2012 | I | 1 | HG008086 | n.d. | HG008112 |
| 12/9773 | Germany | broiler | 2012 | I | 1 | **HG008074** | HG008108 | HG008112 |
| 12/11000 | Denmark | laying chicken | 2012 | I | 1 | HG008086 | HG008108 | HG008111 |
| 12/11056 | Germany | laying chicken | 2012 | I | 1 | **HG008076** | HG008108 | HG008112 |
| 12/11993 | Austria | meat turkey | 2012 | I | 1 | **HG008075** | HG008108 | HG008112 |
| 12/12294 | Hungary | breeders | 2012 | I | 1 | HG008086 | HG008108 | HG008112 |
| 12/12503 | Austria | laying chicken | 2012 | I | 1 | HG008098 | HG008108 | HG008112 |
| 12/13477 | Austria | chicken Sulmtaler | 2012 | I | 1 | **HG008089** | HG008108 | HG008110 |
| 12/15142 | France | turkey breeder | 2012 | I | 1 | AJ920323 | HG008108 | HG008112 |
| 12/17260 | UK | meat turkey | 2012 | I | 1 | **HG008092** | HG008108 | n.d. |
| F-12-18219 | Austria | ostrich | 2012 | I | 1 | HG008086 | n.d. | HG008112 |
| 12/20124 | Netherlands | turkey^#^ | 2012 | I | 1 | HG008098 | HG008108 | HG008112 |
| 13/12607 | Austria | laying chicken | 2013 | II | 1 | HG008095 | HG008107 | HG008109 |
| 13/14913 | France | turkey | 2013 | I | 1 | HG008098 | HG008108 | HG008112 |
| **French isolates collected in 2007-2008** | | | | | | | | |
| 1-06-005/009 | France | turkey breeders | 2007-2008 | II | 5 | HG008095 | HG008107 | HG008109 |
| 2-06-0163/167 |  | turkey breeders | 2007-2008 | I | 5 | HG008093 | HG008108 | HG008110 |
| 3-06-0404/405 |  | turkey breeders | 2007-2008 | II | 2 | **HG008103** | HG008107 | HG008109 |
| 3-06-402 |  | turkey breeders | 2007-2008 | *T. gallinarum* | 1 | HG008103 | n.d. | n.d. |
| 3-06-403/406 |  | turkey breeders | 2007-2008 | *Simplicimonas* spp. | 2 | **HG008104** | n.d. | n.d. |
| 4-07-001/005 |  | meat turkeys | 2007-2008 | I | 5 | **HG008094** | HG008108 | HG008110 |
| 5-07-188/192 |  | meat turkeys | 2007-2008 | I | 5 | **HG008077** | **HG008108** | HG008112 |
| 6-07-465/469 |  | turkey breeders | 2007-2008 | I | 5 | **HG008081** | HG008108 | n.d. |
| 7-07-692/696 |  | meat turkeys | 2007-2008 | I | 5 | AJ920323 | HG008108 | n.d. |
| 8-07-776/780 |  | meat turkeys | 2007-2008 | I | 5 | AJ920323 | HG008108 | n.d. |
| 9-07-1179/1183 |  | meat turkeys | 2007-2008 | I | 5 | **HG008078** | HG008108 | HG008110 |
| 10-07-1393/1397 |  | turkey breeders | 2007-2008 | I | 5 | **HG008079** | HG008108 | **HG008110** |
| 62-3907/3911 |  | meat turkeys | 2007-2008 | I | 5 | HG008105 HG008101 | HG008108 | n.d. |
| 32-975/979 |  | meat turkeys | 2007-2008 | *T, gallinarum*. | 5 | AJ920324 | n.d. | n.d. |
| **French isolates collected in 2009 and 2010** | | | | | | | | |
| 2-1-31/35 | France | meat turkeys | 2009-2010 | I | 4 | **HG008083** | HG008108 | HG008112 |
| 2-1-33 |  | meat turkeys | 2009-2010 | *T. gallinarum* | 1 | HG008102 | n.d. | n.d. |
| 3-1-31/35 |  | turkey breeders | 2009-2010 | I | 5 | **HG008082** | HG008108 | n.d. |
| 4-1/5-31/35 |  | turkey breeders | 2009-2010 | II | 5 | **HG008097** | HG008107 | n.d. |
| 5-1-31/34 |  | turkey breeders | 2009-2010 | I | 4 | **HG008102** | HG008108 | HG008112 |
| 5-1-35 |  | turkey breeders | 2009-2010 | I | 1 | AJ920323 | HG008108 | n.d. |
| 6-2-31 |  | meat turkeys | 2009-2010 | I | 1 | HG008102 | n.d. | n.d. |
| 6-2-32/35 |  | meat turkeys | 2009-2010 | I | 4 | **HG008093** | HG008108 | HG008110 |
| 7-1-31/35 |  | meat turkeys | 2009-2010 | I | 3 | HG008093 | HG008108 | HG008110 |
| 7-1-33/34 |  | meat turkeys | 2009-2010 | *Simplicimonas s*pp. | 2 | HG008105 | n.d. | n.d. |
| 8-1-31/35 |  | meat turkeys | 2009-2010 | I | 5 | HG008079 | HG008108 | n.d. |
| 9-1/6-31/35 |  | meat turkeys | 2009-2010 | II | 28 | HG008095 | HG008107 | HG008109 |
| 9-4-34/ 9-5-32 |  | meat turkeys | 2009-2010 | unknown species | 2 | **HG008100** | n.d. | n.d. |
| 10-1/8-31/35 |  | turkey breeders | 2009-2010 | II | 38 | **HG008096** | HG008107 | HG008109 |
| 10-2-32 10-5-34 |  | turkey breeder | 2009-2010 | *T. gallinarum* | 2 | HG008101 | n.d. | n.d. |
| 11-2/3-31/35 |  | meat turkeys | 2009-2010 | I | 10 | **HG008085** | HG008108 | HG008111 |
| 11-2-35 |  | meat turkeys | 2009-2010 | *T. gallinarum* | 1 | HG008102 | n.d. | n.d**.** |
| 11-4-31/35 |  | meat turkeys | 2009-2010 | I | 4 | HG008104 | HG008108 | **HG008111** |
| 12-1-31/35 |  | meat turkeys | 2009-2010 | I | 5 | AJ920323 | HG008108 | n.d. |
| 13-1/5 |  | meat turkeys | 2009-2010 | I | 5 | **HG008091** | HG008108 | HG008110 |
| 14 |  | meat turkeys | 2009-2010 | *T. gallinarum* | 1 | HG008103 | n.d. | n.d. |
| **Clonal cultures** | | | | | | | | |
| H2 Histomonas meleagridis/Chicken/Hungary/5009-C2/05 | | | | I | 1 | **HG008086 =EU647877** | HG008108 | HG008112 |
| H3 Histomonas meleagridis /Turkey/Austria/2877-C3/05 | | | | I | 1 | **HG008084** | HG008108 | HG008112 |
| H4 Histomonas meleagridis /Turkey/Austria/5642-C4/05 | | | | I | 1 | **HG008080** | HG008108 | HG008112 |
| H7 Histomonas meleagridis /Chicken/Austria/8175-C7/06 | | | | I | 1 | **HG008098** | HG008108 | HG008112 |
| 6B Histomonas meleagridis /Turkey/Austria/2922-C6/04 | | | | I | 1 | **AJ920323** | **FM200068** | **HG008112** |
| H18 Histomonas meleagridis /Turkey/Germany/4114-C18/05 | | | | I | 1 | HG008090 | HG008108 | n.d. |

*n.d .= not determined, PCR amplification was not successful

^$^unknown = type of bird unknown

^#^ type of bird not further determined
